# Improved potential quality of intraoperative transcranial motor-evoked potentials by navigated electrode placement compared to the conventional ten-twenty system

**DOI:** 10.1007/s10143-021-01568-4

**Published:** 2021-05-27

**Authors:** Arthur Wagner, Sebastian Ille, Caspar Liesenhoff, Kaywan Aftahy, Bernhard Meyer, Sandro M. Krieg

**Affiliations:** Department of Neurosurgery, Klinikum Rechts Der Isar, Technische Universität München, Ismaninger Str. 22, 81675 Munich, Germany

**Keywords:** Intraoperative neuromonitoring, Motor-evoked potentials, Potential quality, Navigation

## Abstract

Intraoperative neurophysiological monitoring of transcranial motor-evoked potentials (tcMEPs) may fail to produce a serviceable signal due to displacements by mass lesions. We hypothesize that navigated placement of stimulation electrodes yields superior potential quality for tcMEPs compared to the conventional 10–20 placement. We prospectively included patients undergoing elective cranial surgery with intraoperative monitoring of tcMEPs. In addition to electrode placement as per the 10–20 system, an electrode pair was placed at a location corresponding to the hand knob area of the primary motor cortex (M1) for every patient, localized by a navigation system during surgical setup. Twenty-five patients undergoing elective navigated surgery for intracranial tumors (*n* = 23; 92%) or vascular lesions (*n* = 2; 8%) under intraoperative monitoring of tcMEPs were included between June and August 2019 at our department. Stimulation and recording of tcMEPs was successful in every case for the navigated electrode pair, while stimulation by 10–20 electrodes did not yield baseline tcMEPs in two cases (8%) with anatomical displacement of the M1. While there was no significant difference between baseline amplitudes, mean potential quality decreased significantly by 88.3 µV (− 13.5%) for the 10–20 electrodes (*p* = 0.004) after durotomy, unlike for the navigated electrodes (− 28.6 µV [− 3.1%]; *p* = 0.055). For patients with an anatomically displaced M1, the navigated tcMEPs declined significantly less after durotomy (− 3.6% vs. 10–20: − 23.3%; *p* = 0.038). Navigated placement of tcMEP electrodes accounts for interindividual anatomical variance and pathological dislocation of the M1, yielding more consistent potentials and reliable potential quality.

## Introduction

There exist only few adjuncts in the neurosurgical operating theater as indispensable to the preservation of the patients’ functional integrity as electrophysiological intraoperative neuromonitoring (IONM). The technique was described as early as 1937 and has since been adopted into standard of care for a multitude of scenarios, including the resection of high and low grade gliomas, vascular neurosurgery, as well as spine surgery [5, 6, 8, 12, 17–19]. The premise and principle have remained largely unaltered over the decades: transcranial electrical stimulation of giant pyramidal cells of the primary motor cortex (M1) generates a distinguishable response at the corresponding muscle’s motor unit in the form of a transcranial motor-evoked potential (tcMEP) [2, 3, 15, 16].

It is unsurprising that the technique has also been conjoined with other instruments of surgical planning, primarily with navigational systems facilitating image-guided localization of stimulated motor and functional areas, ultimately providing the operating surgeon with a comprehensive functional concept in any individual case. Despite these sophistications, the anatomical placement of the transcranial stimulation electrodes has not been substantially reviewed since the inception of the conventional *ten-twenty* (10–20) system by Jasper et al. in 1958 for electroencephalography [1, 13].

To our knowledge, no effort has been made to investigate the validity of navigated placement of electrodes for transcranial stimulation of tcMEP. We therefore report on our prospective series of patients undergoing surgery with electrodes placed by both conventional and navigated methods.

We hypothesize that navigated placement of stimulation electrodes reduces the spatial distance between the potential generator (M1) and the potential recorder (the subdermal needle), thus yielding superior potential quality for tcMEPs while applying the same stimulation intensity when compared to the conventional 10–20 placement.

## Methods

### Study design

Patients aged at least 18 years and scheduled for elective cranial surgery were screened for study participation. We included patients undergoing surgery for navigated resection of primary or secondary brain tumors as well as those undergoing navigated resection of vascular lesions, while exhibiting an anatomically displaced or non-displaced M1. Anatomical displacement of the M1 was apprehended on cranial magnetic resonance imaging (MRI) in axial, sagittal, and coronal reconstructions and defined as any spatial variation of the M1 due to a space-occupying lesion, as compared to the contralateral, unaffected side. Patients with lesions that were intrinsic to the M1 or corticospinal tract (CST) were not included, since the centrally located incision would, in these cases, interfere with both 10–20 and navigated tcMEP electrode placements. The use of IONM was routinely indicated in every case.

### Ethical considerations

All procedures were indicated and conducted in compliance with our department’s standards and the Declaration of Helsinki. The local ethics committee granted a positive vote (registration no. 214/16 s), and written informed consent was obtained from all participants before study inclusion.

### Preparation and navigational setup

Preoperative work-up included functional assessment via a neurological examination as well as MRI with diffusion tensor imaging (DTI) and contrast-enhanced T1 and T2 fluid attenuation inversion recovery (FLAIR) sequences. The datasets were made for intraoperative navigational compatibility with an axial slice thickness not exceeding 1 mm.

**Fig. 1:**
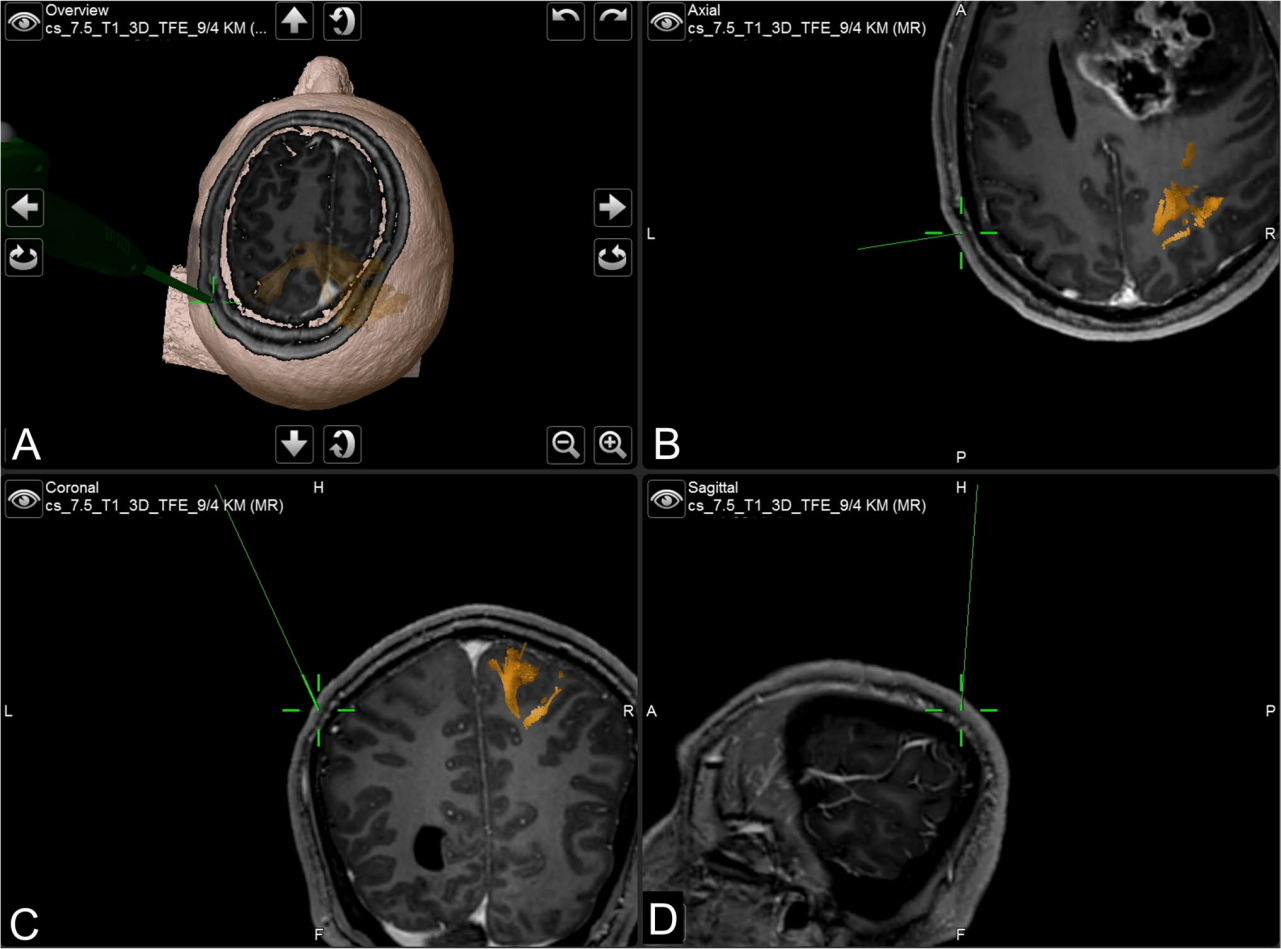
Screenshot of the navigation system. Navigational guide instrument pointing to the left parietal lobe for placement of a transcranial stimulation electrode (**A**). In axial (**B**), coronal (**C**), and sagittal (**D**) reconstructions of a T1-weighted MRI. Corticospinal tract fibers of contralateral side highlighted in yellow according to preoperative functional nTMS mapping

The use of intraoperative navigation guidance was hence mandatory for study inclusion. In addition, preoperative mapping of functional motor areas was conducted for every patient by navigated transcranial magnetic stimulation (nTMS) and visually incorporated into the MRI dataset (Fig. 1).

Patients with implants contraindicative of the use of IONM and those presenting with severe motor deficit (grades < 3 according to the British Medical Research Council (BMRC) grading scale) were not eligible for study inclusion.

### Surgery and neuromonitoring setup

**Fig. 2:**
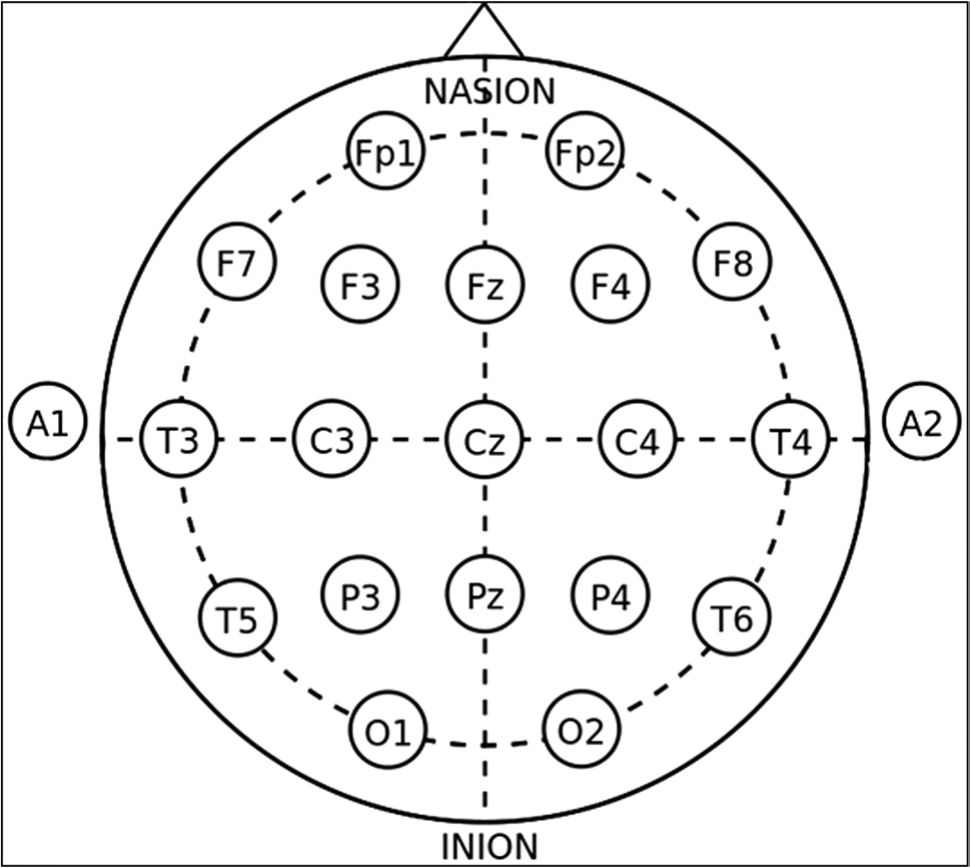
Schematic depiction of the 10–20 system and the electrode placements. C3 and C4 correspond to placements over the M1s

Surgical procedures were not altered for participating patients aside from the additional pair of scalp electrodes placed by navigation. After positioning the patient, a pair of transcranial subdermal needle electrodes (Inomed 27-gauge bipolar needle electrode, Inomed Medizintechnik®) was placed at the C3 and C4 positions in accordance with the 10–20 system and connected to the Inomed ISIS IOM® system (Inomed Medizintechnik®) via a stimulation headbox. The 10–20 scheme inherits its name from the uniform spatial sectioning of the patient’s cranium: halving the distance from the nasion to the inion, the Cz reference point is acquired. From here, partitioning the distance from one tragus to the other in 20% steps results in C3 on the left and C4 on the right side, which indicate the respective cortical projections of the hand area of both M1s, respectively (Fig. 2) [1, 7, 10].

Another pair of subdermal electrodes was then concomitantly placed over both cortical projections of the *hand knobs* as indicated on navigated MRI in the axial, sagittal, and coronal planes with nTMS functional mapping visually superimposed over imaging (Fig. 1). The subdermal needles were inserted into the scalp at a reference point which was closest to the visual representation of the functional area at the M1’s hand knob, with the tip of the needle directed perpendicular to the cortex. The placement of neither the 10–20 nor navigated electrodes was migrated to accommodate for the projected skin incision. After having finalized the placements, the distance between the corresponding electrodes on each side was measured to evaluate the spatial discrepancy between both methods.

Electromyography (EMG) electrodes were placed at key muscles of the upper extremities for every individual in the same fashion: abductor pollicis brevis (APB), abductor digiti minimi (ADM), flexor carpi radialis (FCR), and biceps brachii (BCS). The two separate needles with corresponding cathodal and anodal poles were each positioned with a distance of approximately 10 mm. The electrical impedance was measured continuously throughout the application of stimuli.

**Fig. 3:**
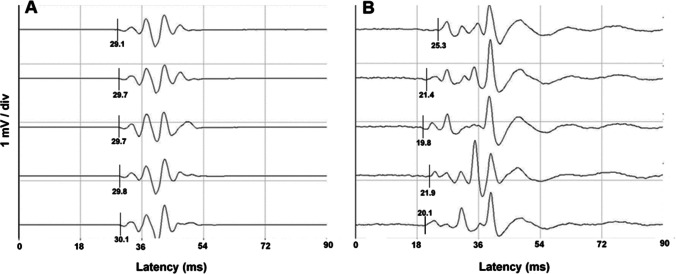
Series of motor-evoked potentials (tcMEPs) in microvolts (µV) of the abductor pollicis brevis muscle for the 10–20 (**A**) and navigated (**B**) placements, respectively

Via the Inomed NeuroExplorer® software (version 5.0, Inomed Medizintechnik®), the initial stimulation settings were preconfigured and adjusted for every patient depending on their individual stimulation threshold. A baseline response was first recorded with a constant-current stimulation intensity of 90 mA, a train of five consecutive monophasic anodal stimuli, each consisting of a 700-µs square-wave pulse and an interstimulus interval of 4.2 ms. The stimulus trains were applied every 10 to 15 s, alternating between the 10–20 and navigated stimulation electrodes, and the resulting tcMEPs were concurrently recorded (Fig. 3). In case of an absent baseline response with the initial stimulation settings, the stimulation intensity was gradually increased in 10-mA increments until a threshold response could be evoked. This baseline threshold intensity was kept unchanged for the remainder of the surgery. Analogously, an excessive motor response to a baseline stimulus of 90 mA led to the tapering of the stimulus intensity in 10-mA decrements until a measurable tcMEP could be recorded without exorbitantly disruptive movements of the extremities. The stimulus intensity was adjusted until the tcMEPs yielded an amplitude strength of 500–2000 µV.

The IONM setup, recordings, and analyses were conducted exclusively by a medical doctor trained in neurophysiology present in the operating theater for the duration of the entire surgery.

The anesthesia consisted solely of continuously infused intravenous agents with propofol and remifentanil as well as rocuronium for intubation. The relaxation effect of rocuronium was gauged by a standard train-of-four stimulation and recording of the response; by the time of setup and stimulation for IONM, the train-of-four responses had returned to normal, signifying subsiding relaxation.

### Follow-up

The patients were followed in accordance with our standard of practice with contrast-enhanced cranial MRI at 3-month intervals. Neurological examinations were conducted on postoperative days 1 and 5, on discharge, and at imaging intervals. Any new onset of a motor palsy or aggravation of an existing deficit by at least 1 on the BMRC scale was recorded as worsening neurological function.

### Statistical analysis

We used IBM SPSS in its 25^th^ version for statistics, and the level of significance was defined a priori as *α* = 0.05. Student’s *t* tests were used for comparison of means and binomial regression analysis for correlation of both the conventional and navigated tcMEP recordings with functional outcome, respectively. The Shapiro–Wilk test was used to test the normal distribution of continuous variables beforehand. The amplitudes of tcMEP recordings were measured from their respective peak to nadir in µV, and the latencies were measured from stimulation to initiation of the tcMEP. Statistical comparisons are based on means generated from the first five recorded tcMEPs of the individual case for each method, respectively. For intergroup comparisons of the relative decreases in amplitudes and latencies, the chi-square test was used. A significant loss of potentials was defined as a reduction in amplitude by at least 50% or an increase in latency by at least 10% as per common conventions [16, 18].

## Results

### Baseline characteristics

We prospectively included 25 consecutive patients undergoing navigated cranial surgery for malignant or vascular lesions between June and August 2019 at our department. Baseline characteristics and postoperative motor status stratified by location of the lesion are depicted in Tables 1 and 2. Stimulation and recording of tcMEPs was successful in every case for the navigated electrodes, while stimulation by 10–20 electrodes did not yield baseline tcMEPs in two cases (8%), with lesions in eloquent locations. One of these eloquent cases had already developed a preoperative motor deficit with a left-sided hemiparesis grade 3, and the other presented with impeccable neurological status. The mean stimulation intensity was 87.6 mA (95% CI 70.0–110.0) for the 10–20 system and 80.8 mA (60.0–100.0; *p* = 0.100) for the navigated electrodes.

**Table 1:** Baseline characteristics of the cohort

Age in years (range)	55.7 (15.38–81.1)
Gender	
Female (%)	40
Entity	
Glioma (%)	68
Metastasis (%)	12
Meningioma (%)	8
Subependymoma (%)	4
AVM (%)	8

**Table 2:** Motor status of the cohort before and after surgery, stratified by anatomical displacement of the M1

M1 displacement	Yes: 48%	No: 52%	*p*^a^
Preoperative motor deficit	33.3%	0%	0.006
Postoperative motor deterioration	8.3%	7.7%	0.110
Intraoperative MEP loss > 50%	8.3%	0%	0.209
Mean spatial distance between the 10–20 and navigated electrodes (cm)	4.1 (− 2.9–11.1)	1.1 (0.8–1.4)	0.203

*M1* primary motor cortex

^a^*p* value of independent *t* test

The distance between 10–20 and navigated electrodes on the lesional side amounted to 1.1 cm (95% CI 0.8–1.4) in patients without anatomical M1 displacement and 4.1 cm (95% CI − 2.9–11.1) with displacement, without a statistically significant difference of spatial distances between these subgroups (*p* = 0.203). In the two cases without obtainable baseline tcMEPs via the 10–20 system, spatial distances were 3.1 cm and 3.4 cm, respectively.

### Amplitudes

**Fig. 4:**
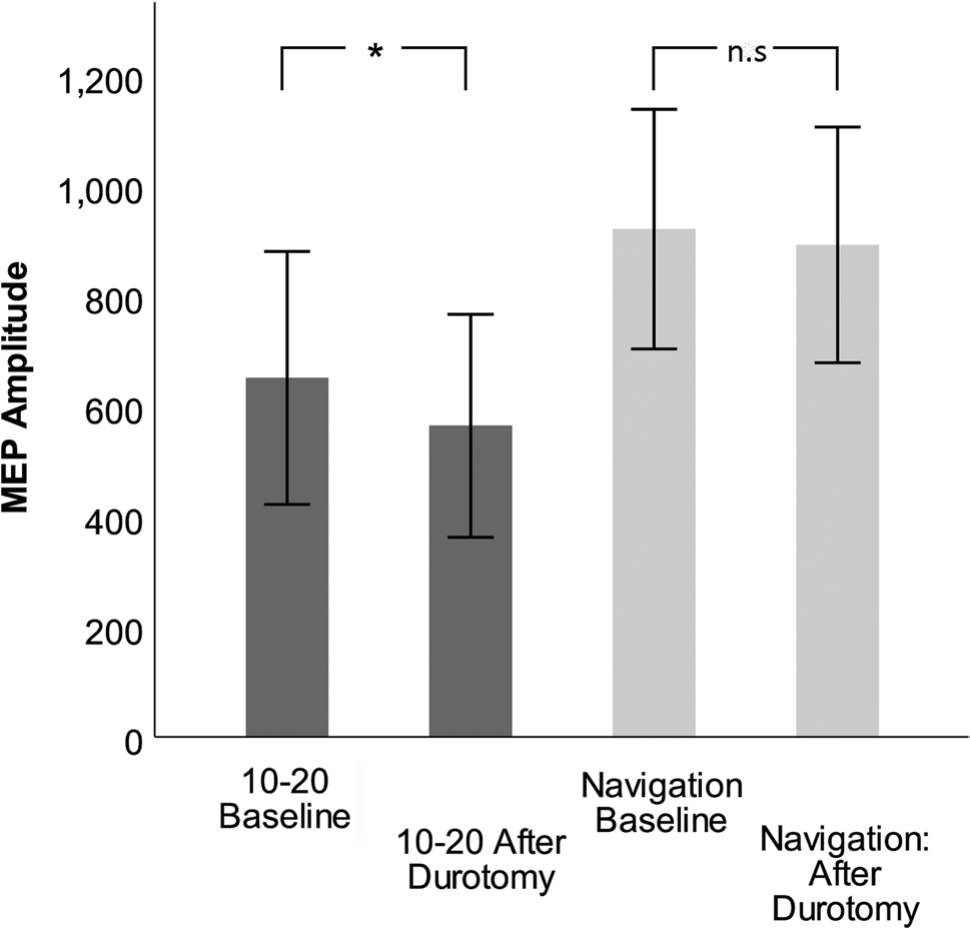
Mean transcranial motor-evoked potentials (tcMEPs) and standard deviations for both the conventional ten-twenty system (10–20) and navigated stimulation at baseline and after dural opening, in the entire cohort. Asterisk indicates statistically significant difference; 10–20 baseline vs. after durotomy: *p* = 0.008; navigation baseline vs. after durotomy: *p* = 0.094

The baseline amplitudes were not significantly different between the 10–20 (672.4 µV) and navigational (866.8 µV; *p* = 0.413) electrodes, although the navigational placement yielded generally higher amplitudes even for cases without M1 displacement (Table 3). Figure 4 depicts mean amplitudes on baseline and after dural opening. In the entire cohort, the mean amplitude decreased significantly by 88.3 µV (− 13.5%) for the 10–20 electrodes (*p* = 0.004) after durotomy, unlike for the navigated electrodes (− 28.6 µV [− 3.1%]; *p* = 0.055). The decrease resulted in a significantly decreased tcMEP in the 10–20 group (584.1 µV [86.5% of baseline]) compared to the navigated group (838.2 µV [96.9% of baseline]; *p* = 0.026).

**Table 3:** Baseline amplitude strengths and latencies of both the conventional ten-twenty (10–20) and navigated stimulation, as well as their respective decreases after dural opening

	M1 displacement		Intergroup comparison, *p*
	No	Yes	
Amplitude			
10–20 baseline (µV)	672.4 (306.5–1038.3)	628.4 (297.7–959.1)	0.271
Navigation baseline (µV)	866.8 (513.2–1220.3)	980.2 (677.7–1282.6)	0.495
Intragroup comparison, *p*	0.413	0.098	
10–20 decrease (µV)	*94.9 (32.9–156.9)*	*146.2 (46.5–245.8)*	0.335
Navigation decrease (µV)	*22.8 (10.6–56.1)*	*35.0 (25.1–87.0)*	0.486
Intragroup comparison, *p*	*0.018*	*0.038*	
Latency			
10–20 baseline (ms)	29.2 (23.3–35.2)	27.3 (21.6–33.1)	0.620
Navigation baseline (ms)	26.5 (21.4–31.7)	29.8 (27.3–32.3)	0.232
Intragroup comparison, *p*	0.462	0.387	
10–20 decrease (ms)	0.5 (0–0.9)	0.3 (− 0.2–0.7)	0.497
Navigation decrease (ms)	0.3 (− 0.5–1.1)	0.9 (− 0.1–2.0)	0.321
Intragroup comparison, *p*	0.351	0.215	

The 95% confidence intervals are in parentheses. Values in italics indicate a statistically significant difference

*M1* primary motor cortex

^a^*p* value of independent and paired *t* tests

When comparing these mean decreases between subgroups of M1 displacement, after durotomy, the potential quality of the navigated electrodes decreased significantly less in patients both with and without M1 displacement (Table 3).

### Latencies

Baseline recordings and changes in latencies are listed in Table 3. Even though latencies measured via navigated stimulations tended to increase more than with 10–20 stimulation, the differences missed statistical significance for both the entire cohort (0.4 ms [± 1.4%, 95% CI 0.1–0.7 ms] vs. 0.6 ms [± 2.1%, 95% CI 0–1.2 ms]; *p* = 0.396) and when stratified for M1 displacement (Table 3).

### Loss of potentials and functional outcome

Postoperative deterioration of motor function was noted in two patients, of which one was found to harbor a right thalamic metastasis without M1 displacement and a preoperative hemiparesis BMRC grade 3. The IONM had predicted a functional decline with a corresponding complete loss of tcMEPs at the conclusion of the resection in this case, while no tcMEP recording was possible by stimulation through the 10–20 electrodes in this case. The signal loss occurred during the final stages of resection, which was consequently halted and the operative site irrigated. The tcMEPs did not return by the end of surgery, and the patient’s hemiparesis deteriorated to a left-sided hemiplegia immediately after. During the postoperative course, however, motor function recovered to BMRC grade 2 for the upper and lower extremities.

In the other case with a right frontal glioma that displaced the M1, motor strength in both contralateral extremities slightly diminished from BMRC grade 5 to a grade 4 palsy, fully recovering on later follow-up. No significant tcMEP losses were registered in this case for either the 10–20 or navigated stimulation or during any other resection, and no deterioration of motor function occurred for any other patient. There were no sequelae or complications directly attributable to the IONM setup or stimulation.

## Discussion

The application of various adjuncts in the operating room is oftentimes hindered or rendered useless through challenges and obstacles imposed by the individual case. Owing to the general complexity of the technique, baseline failure to record any valuable evoked potentials may be imparted by a plethora of causes from various sources; not only may a too rigorous anesthetic induction fully extinguish signals, but minute oversights such as the positioning of cables in relation to sources of electrical noise, the setup of baseline software parameters, or hardware failure of the many components amongst several other sources of error may lead to a failure rate of up to 8% [9, 11, 16]. It is all the more disheartening then to see an immaculate setup still fail to produce any serviceable signal and hence to see a promising surgery be substantially handicapped, most particularly for those difficile lesions in motor eloquent regions. The reason for this unsatisfactory premise generally remains elusive for both neurophysiologist and surgeon, and too few scientific investigations exist for a conclusive approach. Still, it stands to reason that a rigid scheme such as the well-established ten-twenty system that rests on a generalization of intracranial anatomy may not be optimal for the highly individual anatomy of any given case, especially when considering a pathological variant dislodged by a mass lesion. For this reason, the 10–20 grid may obviously fail to produce any serviceable results for the transcranial modality, necessitating the use of a cortical strip electrode [4, 14].

In cases of non-displacing lesions or those near eloquent regions without exposure of the M1, placing a strip electrode is unfeasible; it then follows to optimize the placement of the transcranial electrodes, for which a navigational system seems more than suitable, especially when considering its routine use in most cases of modern tumor resections. In general, striving to reduce the distance between the tip of the stimulation needles and the M1 would result in a reduction of required stimulation intensity for superior sensitivity. This principle is indicated by the higher spatial distance between the corresponding 10–20 and navigated electrodes on the side of the lesion that shifts the M1.

With our investigation, we were able to demonstrate that signal loss was higher for the 10–20 electrodes after durotomy, in both cases with and without M1 displacement, while recording similar baseline amplitudes. This serves our hypothesis that navigated placement of electrodes invariably allows for observation of the individual alterations of a patient’s anatomy and thus is less prone to signal loss even after brain shift and loss of cerebrospinal fluid. An extension of this premise would suggest a lower rate of false-positive tcMEP losses during the course of resection when using navigated stimulation.

The aforementioned limitation of transcranial stimulation in cases of lesions near the M1 was a central presumption of this study. In our experience with these cases, a compromise between conventional electrode placement and surgical access is unavoidable. In essence, the transcranial electrode at the side of the surgical access is often arbitrarily relocated to accommodate for the skin incision, leading to suboptimal or sometimes wholly absent potentials. We specifically opted to adhere to the 10–20 grid for the conventional electrodes and conversely accurately adapt the navigated electrodes to the M1’s (dis)location for the purpose of this study and consistent recordings. The hypothetical superiority in cases of anatomical M1 displacement remains statistically unconfirmed, which may be owed to the significantly inferior amplitude loss for the navigational stimulation in either case. Albeit the difference in tcMEP *stimulation success* missed statistical significance, we maintain that navigated placement may facilitate a serviceable signal where the conventional system fails to do so, particularly when a preexisting motor deficit impedes tcMEP gain.

The latencies we recorded did not exhibit any statistically significant changes for either method, which may be owed to comparably discrete latency changes for both methods and thus a higher case number required.

It was of primary interest to focus on the recording of potentials of the hand and proximal upper extremity to achieve a clean setup with a predefined target for the navigated electrodes. Unsurprisingly, tcMEPs of the lower extremities were absent in a large number of cases. We hypothesize, however, that the utility of the navigated placement may be extrapolated to specifically target the homunculus’ area corresponding to the lower extremities, so that similar results may be achieved.

When considering the prevalent availability of both IONM and navigational systems, little reason is left to sustain and propagate the use of the conventional 10–20 scheme for placement of transcranial stimulation electrodes. With neither additional cost nor preparation time expedited, our results imply a significant benefit of the navigated method, which many practicing surgeons have adopted already. From this, clear clinical utility may be derived for the patients’ postoperative functional independence: the operating surgeon’s assessment of the margin of resection rests on the reliability and validity of the IONM signal. Should this fail to acquire a consistent tcMEP signal, vital information as to the integrity of functional structures is lost, immediately jeopardizing the patient’s postoperative recovery, adjunct therapy regimen, and most importantly, quality of life.

Our study design provides an internal control group, which, according to our data, validates the superiority of navigated electrode placement within predefined anatomic conditions.

Whether navigated electrodes also provide a higher sensitivity and specificity to surgery-induced changes needs to be investigated by a much larger multicentric cohort. Yet, our current data point to this direction showing that such a multicentric approach is worth being investigated.

## Study limitations

This study was designed to compare the validity and reliability of navigated placement of tcMEP electrodes with the conventional technique conforming to the 10–20 scheme. This bears particular interests for those lesions displacing but not invading the M1, as the resection of a lesion within the M1 would require an incision and craniotomy located at C3 and C4, which would certainly interfere with both conventional and navigated tcMEP electrode placements. As a result, patients with lesions within the M1 were excluded from this study, although further investigations may be designed to include a comparative group being monitored by a cortical strip electrode. Still, the navigational placement of tcMEP electrodes is specifically intended to precisely target M1 areas of interest, especially when the 10–20 technique fails to account for anatomical displacement. Despite our data pointing to superior potential quality of the navigated tcMEPs, we maintain that cortical and subcortical stimulation should still be used for resections within the M1.

Analogously, the clinical utility may have been emphasized by a control group without navigated tcMEP monitoring. The study was designed with an internal control, hence both techniques were applied concurrently and any feedback to the operating surgeon would have prompted a change of surgical strategy, such as in one case with signal loss. This notion precludes differentiation of the surgery-altering consequences of the individual techniques, which would require said control group without concurrent monitoring. In a cohort with a higher rate of postoperative motor deterioration and tcMEP signal loss, a clearer difference between both techniques might have been emerged. An important caveat of all these assumptions may be found in the possible discrepancy between the anatomical hand knob area and the localization of its functionality, although we accounted for this by preoperative nTMS mapping of functionality and incorporated its visualization into the needle placement. Our results appear plausible and with a distinct advantage for the navigational stimulation, although the sample size is fairly small and warrants further investigation in larger controlled studies, before this advantage can be generalized.

## Conclusion

Due to its adherence to a rigid scheme, placement of transcranial stimulation electrodes via the conventional 10–20 system may yield significantly less durable potential quality, regardless of whether there is mass effect displacing the M1. Navigated placement is quick, safe, and tailored to each patient’s anatomic characteristics and should be favored over the traditional placement if neuronavigation is available.

## Data Availability

Data are available on reasonable request to the corresponding author.
